# Simulation of Eccentric Impact of Square and Rectangular Composite Laminates Embedded with SMA

**DOI:** 10.3390/ma11122371

**Published:** 2018-11-26

**Authors:** Min Sun, Mengzhou Chang, Zhenqing Wang, Hao Li, Yanfei Liu

**Affiliations:** 1 College of Aerospace and Civil Engineering, Harbin Engineering University, Harbin 150001, China; sunmin@hrbeu.edu.cn (M.S.); changmengzhou@hrbeu.edu.cn (M.C.); Lihao0202@hrbeu.edu.cn (H.L.); 2 State Key Laboratory of Tribology, Tsinghua University, Beijing 100084, China; yanfeiliu@hrbeu.edu.cn

**Keywords:** eccentric impact, finite element analysis, shape memory alloy (SMA) wire, composite laminates

## Abstract

In the present work, we study the low velocity impact, both central and eccentric, on square and rectangular laminated composite plates with embedded shape memory alloy (SMA) wires, which are stitched on the top and bottom surfaces of the plate, by using the finite element method. In finite element methods (FEM) simulations, a super-elastic SMA constitutive model is implemented in Abaqus/Explict by using a user defined material subroutine to describe the behaviors of SMAs. The three-dimensional (3D) Hashin failure criterion is adopted to model the damage initiation of laminated composite plates. To model the delamination failure, a cohesive damage zone model is introduced in interface elements. A comprehensive parametric study has been carried out to analyze the effects of eccentricity for the case of square and rectangular laminated composite plates.

## 1. Introduction

Composite materials are widely used in aerospace structures, however, they are usually vulnerable to accidental and eccentric impacts from various multitude and complex loading conditions, such as dropped tools, gravels collision, bird strike, hails and so on. Thus, the study of the low velocity impact behavior of laminated composite plates is a very important task.

In the last several decades, a few experimental and numerical investigations had been carried out to study the response of SMA-reinforced laminated composite plates subjected to low velocity central impact [1,2,3,4,5,6,7,8,9,10]. However, the study on low velocity eccentric impact are still limited. So far, the study of low velocity eccentric impact has only been reported by Shariyat et al. [11,12,13,14] in literature. First of all, they only investigated the low velocity eccentric impact analysis of rectangular laminated composite plates subjected to in-phase/anti-phase biaxial preloads (see reference [11]). In their work, they presented a nonlinear finite element formulation to simulate a low velocity eccentric impact between a rigid spherical indenter and a laminated composite rectangular plate with asymmetric lamination structure. By considering the different contact laws for the loading and unloading phases, they investigated the effect of parameters (the specifications of the plates and the indenter, the eccentric value, and the in-plane preloads) on the indentation and force time histories. It was found that the compressive preloads in-plane will reduce the contact force (that is indentation values), the tensile preloads in-plane will increase the contact force, and the extensile tensile preloads may lead to higher damages. Moreover, they also investigated the low velocity eccentric impact analysis of transversely graded plates with Winkler-type elastic foundations and fully/partially supported edges (see reference [12]). In their work, they make contributions in numerical simulation by taking into account the contact law, the elastic foundation, the material heterogeneity, partially supporting the edges, and semi-analytical solutions. The major novelty of their approach is a novel double superposition power-exponential global-local theory and a refined contact law to investigate low velocity eccentric impact responses of rectangular sandwich plates with viscoelastic cores (see reference [13]). In this work, they investigated effects of the pre-stresses on the indentation and contact force, and the effects of the eccentricity on the impact responses of the sandwich plates for the first time. The results showed that the contact force and the absorbed energy will increase, and the failure will more likely occur in the low velocity eccentric impact. Furthermore, they further analyzed the accurate eccentric impact for composite plates with embedded the preloaded SMA based on a novel mixed-order hyperbolic global-local theory (see reference [14]). In their work, they propose a higher-order global-local hyperbolic plate theory including both odd and even functions to describe the general asymmetric displacement fields; and moreover, Shariyat and his co-workers have considered some other factors, such as the non-uniform and time-dependent distributions of the phases of the SMA wires and different contact laws for loading and unloading phases.

Although some efforts have been made for studying the response of the eccentric impact on composite plates, the scope of investigations is still limited. Moreover, a comprehensive analysis for the low velocity eccentric impact of the SMA reinforced laminated composite plates is still lacking. Therefore, in the present work, we shall reexamine the low velocity impact, both central and eccentric, on square and rectangular laminated composite plates with embedded SMA wires, which are stitched on the top and bottom surfaces of the plate. In the present study, the numerical simulation is carried out by using Abaqus/Explict finite element software with version 6.14, dassault SIMULIA Inc, Providence, RI, USA. The paper is arranged four sections. The constitute model of SMA wires, the constitute laws of laminated composite plates, the cohesive zone model between SMA and plates, and the failure criterion of composite plates are discussed in Section 2. In Section 3, the modeling framework of the SMA reinforced square and rectangular laminated composite plate under low velocity central and eccentric impact are given. In Section 4, the impact responses of the rigid spherical indenter for the cases of low velocity central and eccentric impact are analyzed, and they are compared with the results of Shariyat et al. (see [14]). Moreover, the damage morphology patterns of SMA reinforced square/rectangular laminated composite plates are also analyzed, and the lateral deflection histories of different impact points in the top/bottom layer SMAs and the composite plates are discussed. Finally, we conclude the study in Section 5 with a few conclusions drawn.

## 2. The Governing Equations

### 2.1. The Constitutive Model of SMA Wires

**Figure 1:**
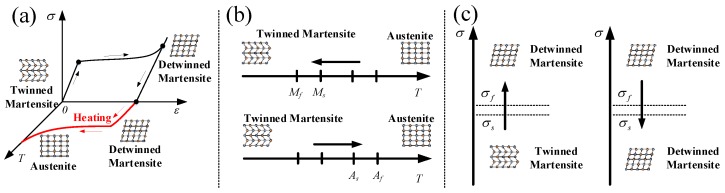
Schematic diagrams of phase transformation of typical Ni-Ti SMAs corresponding to the crystal structures: (**a**) stress-strain-temperature curve; (**b**) temperature-induced phase transformation; (**c**) stress-induced phase transformation.

SMAs have two phases (see Figure 1a) of austenite (A) and martensite (M) which exists in two forms of twinned martensite and detwinned martensite, thus different phases have different crystal structure and different properties. Furthermore, the properties of SMAs are associated with the stress-induced (see Figure 1c) and temperature-induced (see Figure 1b) phase transformation. Therefore, the forward transformation from austenite to martensite and the reverse transformation from martensite to austenite form the unique behavior (such as super-elasticity, shape memory effect, and dissipated energy effect) of SMAs.

In recent decades, various constitutive models of SMA have been presented, however, Brinson’s model [15] is most often referred to in study. Therefore, in the present paper, the stress-strain relation of SMA based on Brinson’s constitutive equation can be denoted as

(1) $$\sigma -\sigma_{0}=D\left(\varepsilon -\varepsilon_{0}\right)+\Omega \left(\xi_{S}-\xi_{S_{0}}\right)+\Omega \left(\xi_{T}-\xi_{T_{0}}\right)+\Theta \left(T-T_{0}\right)$$

where σ is the Cauchy stress tensor ($\sigma_{0}=0$), ε is the infinitesimal strain tensor ($\varepsilon_{0}=0$), D is the modulus which can be assumed to be a function of martensite fraction ($D=D_{a}+\xi \left(D_{m}-D_{a}\right)$), which $D_{m}$ and $D_{a}$ are Young’s modulus of SMA for a pure martensite and austenite, respectively), $\xi_{S}$ is the purely stress-induced martensite fraction ($\xi_{S_{0}}=0$), $\xi_{T}$ is the purely temperature-induced martensite fraction ($\xi_{T_{0}}=0$), Ω is the transformation tensor (Ω=−ξD), and Θ is tangent expansion modulus tensor which is related to the thermal expansion coefficient of SMA material, T is temperature of SMA ($T_{0}$ is the reference temperature). The properties of SMAs are associated with phase transformation, thus the martensite fraction equation in accordance with the stress and temperature variation can be defined as the following three regions [16,17]:

(i) Conversion from the austenite to the detwinned martensite phase for $T>M_{s}$ and $\sigma_{s}^{cr}+C_{M}\left(T-M_{s}\right)<\sigma <\sigma_{f}^{cr}+C_{M}\left(T-M_{s}\right)$:

(2-1) $$\xi_{S}=\frac{1-\xi_{S_{0}}}{2}\cos\left\{\frac{\pi }{\sigma_{s}^{cr}-\sigma_{f}^{cr}}\left(\sigma -\sigma_{f}^{cr}-C_{M}\left(T-M_{s}\right)\right)\right\}+\frac{1+\xi_{S_{0}}}{2}$$

(2-2) $$\xi_{T}=\xi_{T_{0}}-\frac{\xi_{T_{0}}}{1-\xi_{S_{0}}}\left(\xi_{S}-\xi_{S_{0}}\right)$$

(ii) Conversion from the austenite to the detwinned martensite phase for $T<M_{s}$ and $\sigma_{s}^{cr}<\sigma <\sigma_{f}^{cr}$:

(3-1) $$\xi_{S}=\frac{1-\xi_{S_{0}}}{2}\cos\left\{\frac{\pi }{\sigma_{s}^{cr}-\sigma_{f}^{cr}}\left(\sigma -\sigma_{f}^{cr}\right)\right\}+\frac{1+\xi_{S_{0}}}{2}$$

(3-2) $$\xi_{T}=\xi_{T_{0}}-\frac{\xi_{T_{0}}}{1-\xi_{S_{0}}}\left(\xi_{S}-\xi_{S_{0}}\right)+\Delta_{T\xi }$$

where,

(3-3) $$\Delta_{T\xi }=\left\{ \begin{array}{l} \frac{1-\xi_{T_{0}}}{2}\left[\cos\left(\frac{\pi }{M_{s}-M_{f}}\left(T-M_{f}\right)\right)+1\right],\;M_{f}<T<M_{s}\;\mathrm{and}\;T<T_{0}\\ 0,\;other \end{array}\right.$$

(iii) Conversion from the detwinned martensite to the austenite phase for $T>A_{s}$ and $C_{A}\left(T-A_{f}\right)<\sigma <C_{A}\left(T-A_{s}\right)$:

(4-1) $$\xi =\frac{\xi_{0}}{2}\left\{\cos\left[\frac{\pi }{A_{f}-A_{s}}\left(T-A_{s}-\frac{\sigma }{C_{A}}\right)\right]+1\right\}$$

(4-2) $$\xi_{S}=\xi_{S_{0}}-\frac{\xi_{S_{0}}}{\xi_{0}}\left(\xi_{0}-\xi \right)$$

(4-3) $$\xi_{T}=\xi_{T_{0}}-\frac{\xi_{T_{0}}}{\xi_{0}}\left(\xi_{0}-\xi \right)\;$$

where $M_{s}$ is the martensitic start temperature at zero stress, $M_{f}$ is the martensitic finish temperature at zero stress, $A_{s}$ is the austenitic start temperature at zero stress, $A_{f}$ is the austenitic start temperature at zero stress, $C_{M}$ is the stress influence coefficient of martensite (which is also the slope of the martensite transformation curve), $C_{A}$ is the stress influence coefficient of austenite (which is also the slope of the austenite transformation curve), $\sigma_{s}^{cr}$ is the initiation critical stress for forward transformation into martensite, $\sigma_{f}^{cr}$ is the finish critical stress for forward transformation into martensite. In the FE simulation, the material model of SMAs is implemented by using a user-defined subroutine VUMAT that is adopted in Abaqus/Explicit with version 6.14, dassault SIMULIA Inc, Providence, RI, USA. Furthermore, the specific material properties of Ni-Ti SMA wires are listed in Table 1. 

### 2.2. Constitutive Laws of Fiber Reinforced Composites

Glass fiber and the matrix used in this paper are regarded as homogeneous isotropic materials on microscale. The constitutive model can be denoted as:

(5) $$\sigma_{ij}=2G\varepsilon_{ij}+\lambda \varepsilon_{kk}\delta_{ij}$$

where, $\sigma_{ij}$ and $\varepsilon_{ij}$ are nominal stress and strain (*i* = *j*), shear stress and strain (*i* ≠ *j*), respectively, here, (*i*, *j* = x, y and z are the reference coordinates X). Besides, $\lambda =E\frac{\mu }{\left(1+\mu \right)\left(1-2\mu \right)}$, $G=\frac{E}{2\left(1+\mu \right)}$, $\varepsilon_{kk}=\varepsilon_{xx}+\varepsilon_{yy}+\varepsilon_{zz}$ and $\delta_{ij}=1,\;(i=j)\;;\delta_{ij}=0,\;(i\neq j)$ are the Lame’s constant, shear modulus, volumetric strain, and Kronecker delta, respectively. E and μ are the elastic modulus and Poisson’s ratio. The strain field $\varepsilon_{ij}=\frac{1}{2}\left(\frac{\partial u_{i}}{\partial X_{j}}+\frac{\partial u_{j}}{\partial X_{i}}\right)$, $u_{i}$ is the displacement field. Furthermore, the stress strain relationship of composites in undamaged state can be rewritten as:

(6) $$\left\{\begin{array}{l} \sigma_{11}\\ \sigma_{22}\\ \sigma_{33}\\ \sigma_{23}\\ \sigma_{31}\\ \sigma_{12} \end{array}\right\}=\left\{\begin{array}{cccccc} c_{11} & c_{12} & c_{13} & 0 & 0 & 0\\ c_{21} & c_{22} & c_{23} & 0 & 0 & 0\\ c_{31} & c_{32} & c_{33} & 0 & 0 & 0\\ 0 & 0 & 0 & c_{44} & 0 & 0\\ 0 & 0 & 0 & 0 & c_{55} & 0\\ 0 & 0 & 0 & 0 & 0 & c_{66} \end{array}\right\}\left\{\begin{array}{l} \varepsilon_{11}\\ \varepsilon_{22}\\ \varepsilon_{33}\\ \varepsilon_{23}\\ \varepsilon_{31}\\ \varepsilon_{12} \end{array}\right\}\;$$

where, $c_{ij}$ are the stiffness coefficients which can be derived from G and λ. At elastic state, the specified damage variables $d_{i}$ are equal to 0. The constitutive laws of glass fiber composites are implemented in Abaqus/Explicit by using a user-defined subroutine (VUMAT), and the material properties of the glass fiber-epoxy laminates are shown in Table 2.

### 2.3. Interlaminar Damage Model

In the paper, the delamination between interlayer interfaces or SMA and layer interfaces are modelled by surface-based cohesive behavior in Abaqus/Explicit. The surface-based cohesive behavior is defined as a surface interaction property and can be used to model the delamination at interfaces directly in terms of traction versus separation [18,19,20,21]. The available traction-separation model in Abaqus assumes initially linear elastic behavior, followed by the initiation and evolution of damage. The elastic behavior is written in terms of an elastic constitutive matrix that relates the normal and shear stresses to the normal and shear separations across the interface. The elastic behavior can then be written as

(7) $$t=\left\{\begin{array}{c} t_{n}\\ t_{s}\\ t_{\tau } \end{array}\right\}=\left[\begin{array}{ccc} K_{nn} & 0 & 0\\ 0 & K_{ss} & 0\\ 0 & 0 & K_{tt} \end{array}\right]\left\{\begin{array}{c} \delta_{n}\\ \delta_{s}\\ \delta_{t} \end{array}\right\}=Κ\delta$$

where, nominal traction stress vector t, consists of three components $t_{n}$, $t_{s}$ and $t_{t}$, which represent the normal and two shear tractions, respectively. Here, we adopt the uncoupled traction-separation behavior, and the terms $K_{nn}$, $K_{ss}$ and $K_{tt}$ are not defined any dependencies on temperature or field variables. Abaqus uses default contact penalties to model the traction-separation behavior.

**Figure 2:**
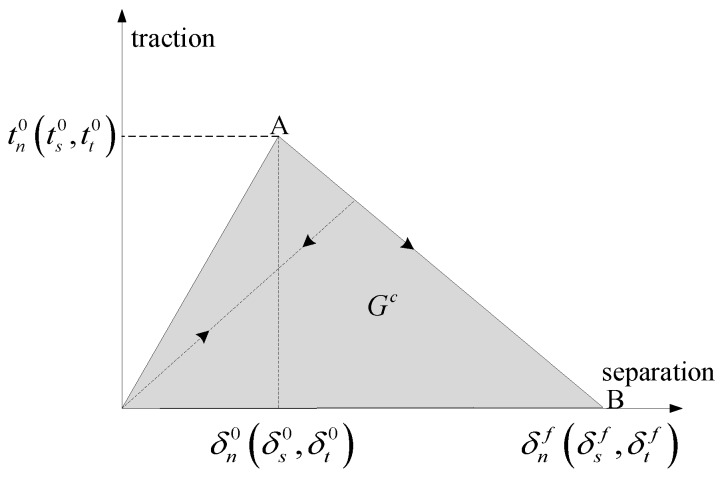
Typical traction-separation response.

Damage modeling simulate the degradation and eventual failure of the bond between two cohesive surfaces. The failure mechanism consists of two ingredients: A damage initiation criterion and a damage evolution law. The initial response is assumed to be linear, and once a damage initiation criterion is met, damage can occur according to a user-defined damage evolution law. Figure 2 shows a typical traction-separation response with a failure mechanism.

Damage initiation refers to the beginning of degradation of the cohesive response at a contact point. The process of degradation begins when the contact stresses satisfy certain damage initiation criteria. Damage is assumed to initiate when a quadratic interaction function involving the contact stress ratios reaches a value of one. This criterion can be represented as:

(8) $${\left\{\frac{t_{n}}{t_{n}^{0}}\right\}}^{2}+{\left\{\frac{t_{s}}{t_{s}^{0}}\right\}}^{2}+{\left\{\frac{t_{t}}{t_{t}^{0}}\right\}}^{2}=1$$

where, $t_{n}^{0}$, $t_{s}^{0}$ and $t_{t}^{0}$ represent the peak values of the contact stress when the separation is either purely normal to the interface or purely in the first or the second shear direction, respectively.

The damage evolution law describes the rate at which the cohesive stiffness is degraded once the corresponding initiation criterion is reached. Damage evolution can be defined based on the energy that is dissipated as a result of the damage process, also called the fracture energy. The fracture energy is equal to the area under the traction-separation curve in Figure 2. Unloading subsequent to damage initiation is always assumed to occur linearly toward the origin of the traction-separation plane, as shown in Figure 2. Reloading subsequent to unloading also occurs along the same linear path until the softening envelope (line AB) is reached. Once the softening envelope is reached, further reloading follows this envelope as indicated by the arrow in Figure 2. The dependence of the fracture energy on the mode mix is defined based on a power law fracture criterion. The power law criterion states that failure under mixed-mode conditions is governed by a power law interaction of the energies required to cause failure in the individual (normal and two shear) modes. It is given by:

(9) $${\left\{\frac{G_{n}}{G_{n}^{c}}\right\}}^{\alpha }+{\left\{\frac{G_{s}}{G_{s}^{c}}\right\}}^{\alpha }+{\left\{\frac{G_{t}}{G_{t}^{c}}\right\}}^{\alpha }=1$$

where, where α =1, and $G_{n}$, $G_{s}$ and $G_{t}$ are the work done by traction and its conjugate relative displacement in the normal and two shear directions, respectively. $G_{n}^{c}$, $G_{s}^{c}$ and $G_{t}^{c}$ refer to the critical fracture energies required to cause failure in the normal, the first, and the second shear directions, respectively. In the present paper, the relevant cohesive parameters values adopted in FE simulation are as follows [22]: $K_{nn}=K_{ss}=K_{tt}=3.9\;\mathrm{GPa}/m$, $\sigma_{n}^{0}=97.5\;\mathrm{MPa}$, $\tau_{s}^{0}=\tau_{t}^{0}=39\;\mathrm{MPa}$, and $G_{n}^{c}=10\;N/m,\;G_{s}^{c}=G_{t}^{c}=90\;N/m$.

### 2.4. Failure Criterion

Plane stress is a significant parameter to estimate the stress state of laminated composite plate, and the failure criterion is necessary to predict the failure of plate under combined stress states. In the last decades, the 3D Hashin failure criterion is the most often criterion used in study. Therefore, the three-dimensional failure criterion based on Hashin failure model are described as follows [23,24,25]:

Fiber tension failure ($\sigma_{11}>0$)

(10-1) $${\left(\frac{\sigma_{11}}{X_{T}}\right)}^{2}+{\left(\frac{\sigma_{12}}{S_{12}}\right)}^{2}+{\left(\frac{\sigma_{13}}{S_{13}}\right)}^{2}\geq 1$$

Fiber compression failure ($\sigma_{11}<0$)

(10-2) $${\left(\frac{\sigma_{11}}{X_{C}}\right)}^{2}\geq 1$$

Matrix tension failure ($\sigma_{22}+\sigma_{33}>0$)

(10-3) $${\left(\frac{\sigma_{11}}{2X_{T}}\right)}^{2}+{\left(\frac{\sigma_{12}}{S_{12}}\right)}^{2}+\frac{{\left(\sigma_{22}\right)}^{2}}{Y_{T}Y_{C}}+\frac{\sigma_{22}}{Y_{T}+Y_{C}}\geq 1$$

Matrix compression failure ($\sigma_{22}+\sigma_{33}<0$)

(10-4) $${\left(\frac{\sigma_{11}}{2X_{T}}\right)}^{2}+{\left(\frac{\sigma_{12}}{S_{12}}\right)}^{2}+\frac{{\left(\sigma_{22}\right)}^{2}}{Y_{T}Y_{C}}+\frac{\sigma_{22}}{Y_{T}+Y_{C}}\geq 1$$

where $X_{T}$, $X_{C}$, $Y_{T}$ and $Y_{C}$ are the tensile and compressive strengths in the longitudinal and transverse directions, respectively, and $\sigma_{ij}$ (*i*, *j* = 1, 2, 3) are the Cauchy stress tensor components. $S_{12}$ is the shear strength in the fiber and transverse direction, $S_{13}$ is the shear strength in the fiber and thickness direction, and $S_{23}$ is the shear strength in the transverse and thickness direction, respectively.

In the present paper, the failure criterion is modelled by using a user-defined subroutine (VUMAT) that is adopted in Abaqus/Explicit to analyze the damage mechanisms of laminated composite plate. The relevant strength parameters adopted in FE simulation are listed in Table 3. It should be noted that the failure elements will be eliminated from geometry and will be not considered in further calculations in order to ensure the stability during analysis.

The failure criterion of SMA adopts the theory of maximum tensile stress. When the maximum tensile stress of the material reaches a certain limit value (that is, the strength limit measured by the axial tensile test of material), the material breaks and the strength formula is as follows:

(10-5) $$\sigma_{1}\leq \left[\sigma \right]$$

where $\sigma_{1}$ is the maximum tensile stress of the material, [σ] is the strength limit of the material.

## 3. Modeling Framework

**Figure 3:**
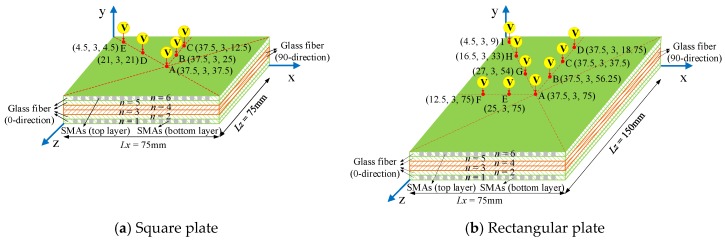
Schematic diagrams of the SMA reinforced square and rectangular laminated composite plate.

In the present paper, the SMA reinforced square laminated composite plate has the dimension $L_{x}\times L_{y}\times L_{z}$ = 75 mm × (n × 0.5 mm) × 75 mm, and the SMA reinforced rectangular laminated composite plate has the dimension $L_{x}\times L_{y}\times L_{z}$ = 75 mm × ( n × 0.5 mm) × 150 mm, in which n = 6 is the ply number of glass fiber in plate, and the whole thickness of plate is 3 mm. The stacking sequence of square and rectangular laminated composite plates are both [0_2_/90_2_/0_2_], and the SMA wires which parallel to the 0° glass fiber direction are embedded in the top and bottom layer surfaces of the laminated composite plates (see Figure 3). Figure 3 show the schematic diagrams of geometric parameters and stacking sequences of the SMA reinforced square and rectangular laminated composite plate. The SMAs adopt the square section, and the height of square SMA wires is equal to the layer thickness of glass fiber, which is 0.5mm. For the case of the square composite plate, the number of SMA wires stitched in plate is 45 roots, and the length of each root is 75 mm. For the case of the rectangular composite plate, the number of SMA wires stitched in plate is also 45 roots, and the length of each root is 150 mm. The interval spacing between two SMA wires is both 1.5 mm for these two cases of square and rectangular composite plates. In the present study, the model setting and related conditions of SMA reinforced square laminated composite plate are consistent with the literature [14].

**Figure 4:**
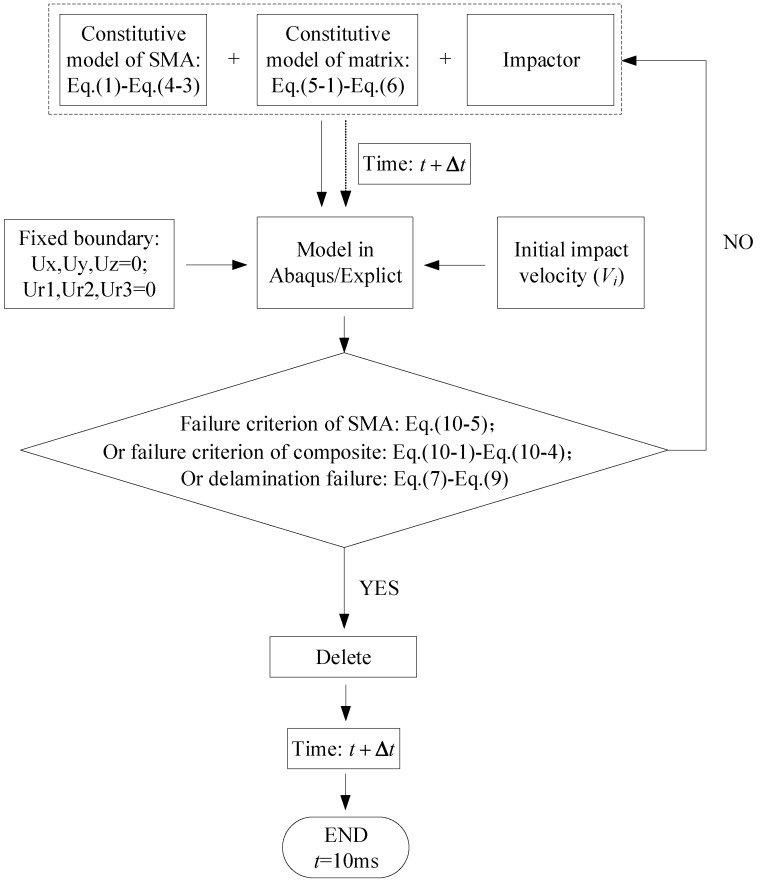
Flow chart for numerical implementation of constitutive model and failure criterion.

Figure 4 shows a flow chart of a finite element numerical model based on the constitutive model and the failure criteria. Firstly, the numerical model is established in ABAQUS/explicit based on the actual sizes of the SMA wires, composite laminate and impactor, which the three are modeled by solid elements. Secondly, the model is attached with the basic parameters corresponding to the actual material. Thirdly, the model is set to the failure criterion of SMA wire, the in-layer and inter-layer failure criterion of composite material, and the failure criterion between SMA wire and laminate interface. Fourthly, the various models are meshed, and then are assembled. Finally, the boundary conditions of the whole model are set, and the initial impact velocity is given according to the actual situation.

**Figure 5:**
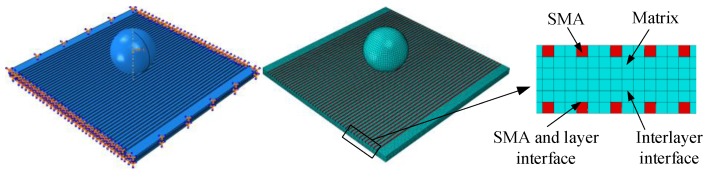
Finite element model of composite laminate under impact loading.

The finite element model of SMA reinforced composite laminates is generated and analyzed by ABAQUS/Explicit. Figure 5 shows the finite element model of the laminate under impact loading. The four sides of the laminate are fixed, and the impact point is located at the center of the laminate. Fiber and resin are considered as a whole, and SMA wires are used as a reinforcement, both are meshed using an 8-node linear brick, reduced integration, hourglass control (C3D8R). The laminate is finely meshed with elements 0.5 mm × 0.5 mm in size, and the SMA wire is finely meshed with elements 0.5 mm × 0.5 mm in size. The mesh density in laminate and SMA wire are chosen on the basis of a sensitivity analysis that shows convergence of solutions considering both the structural response and the internal damage when using element sizes smaller than approximately 1 mm. The impactor is seen as a rigid sphere and meshed by R3D4 rigid unit. The radius, mass, initial velocity, and initial energy of the rigid spherical indenter are 9.6 mm, 3 kg, 5 m/s, and 37.5 J. 

## 4. Results and Discussions

### 4.1. Verification of the Results

**Figure 6:**
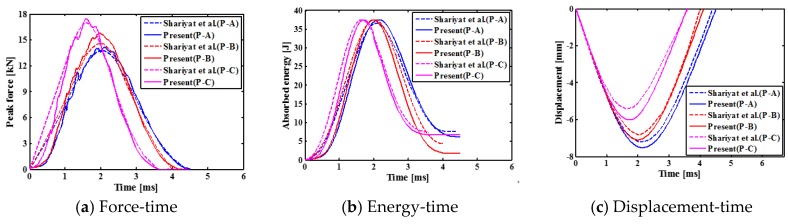
Comparison between present numerical impact results and results of Shariyat et al. [14].

First of all, the numerical analysis of SMA reinforced square laminated composite plate is carried out for the case of low velocity central impact and eccentric impact with rigid spherical indenter. Comparing present results with those of Shariyat et al. [14] in Figure 6, it is found that it has good agreement between these results. Figure 6 shows the contact force histories, the absorbed energy histories and the displacement histories of indenter of the SMA reinforced square laminated composite plate. As seen from Figure 6, the contact time between indenter and plate during the impact process is shorter for the cases of eccentric impact (Point B and C) than the case of central impact (Point A). Moreover, for the cases of eccentric impact (Point B and C), the peak forces of indenters are larger, and the displacements of indenters are smaller. This is mainly due to the impact point is the farthest point from the boundary to impact center of laminate under center impact. As the distance decreases, the stiffness of the laminate increases. Furthermore, the specific values of the impact parameters are also listed in Table 4.

### 4.2. Low Velocity Impact of SMA Reinforced Square Laminated Composite Plates

#### 4.2.1. Typical Impact Curves of Indenter

**Figure 7:**
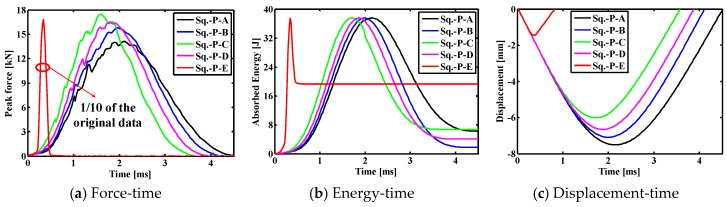
Comparison results between different impact positions of the SMA reinforced square laminated composite plates with stacking sequences [SMA/0_2_/90_2_/0_2_/SMA].

Figure 7 shows the comparison results for the contact force histories, the absorbed energy histories and the displacement histories of indenter of the SMA square laminated composite plate with stacking sequences [SMA/0_2_/90_2_/0_2_/SMA] for the cases of the different impact positions. As can be seen, the contact time between the impactor and the laminate is the longest, the maximum contact force value is the smallest, the displacement value is the largest under the center contact (point A). The contact time between the impactor and the laminate is shorter, the maximum contact force value is larger, and the displacement value is smaller under the eccentric impact (point B/C/D). The contact time between the impactor and the laminate is the shortest, and the maximum contact force value is the maximum, the displacement value is the smallest under the eccentric impact (point E). From the perspective of the above parameters, it can be seen that reducing the distance of impact point to the boundary is beneficial to increase the impact resistance of the entire laminate. In addition, the final absorbed energy value from large to small is followed by the condition with impact point E, point C, point A, point D, point B. Comparing point A with point B, it can be seen that as the distance from the impact point to the boundary decreases, the final absorption energy of the laminate decreases, and the recoverable absorption energy of the entire plate increases. Comparing point B with point D, it can be seen that as the distance from the impact point to the boundary decreases, the final absorption energy of the laminate increases, and the recoverable absorption energy of the entire plate decreases. That is to say, a reasonable eccentric impact position is beneficial to reduce the final absorption energy of the laminate and thereby increase the recoverable absorption energy of the entire plate. For the square laminates simulated in this paper, this position is between points B and D, that is, the distance between 21 and 25 mm.

In order to more clearly illustrate the peak contact force, the final absorbed energy, and the maximum displacement, the specific values of the impact parameters under different impact positions in a square composite laminate are listed in Table 5. Under the central impact (point A), the peak contact force, final absorption energy and maximum displacement of the composite laminate are 14.14 kN, 6.17 J and −7.51 mm, respectively. Under the eccentric impact (B/C/D/E point), the peak contact forces of composite laminates are 15.79, 17.48, 16.47 and 168.07 kN, respectively, and the final absorption energies are 1.79, 6.74, 4.04 and 19.25 J, respectively, the maximum displacements are −7.10 mm, −6.01 mm, −6.66 mm and −1.45 mm, respectively. For the eccentric impact of point E, the peak contact force, the final absorbed energy and the maximum displacement value are quite different from that of other eccentric impact points. This is mainly because the distance from the impact point to the boundary is smaller than the impactor radius, and the impactor rebound occurred during the process.

#### 4.2.2. Damage Patterns of SMA Reinforced Square Laminated Composite Plate

**Figure 8:**
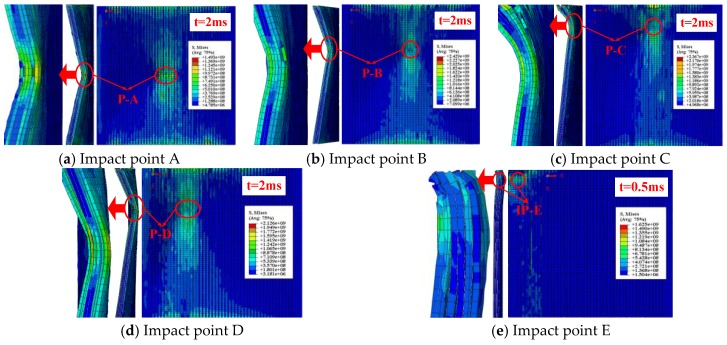
Impact contact surface and cross section damage patterns of SMA reinforced square laminated composite plate subjected to low velocity central impact (Point A) and eccentric impact (Point B/C/D/E).

Figure 8 shows the impact contact surface and the cross section damage patterns of SMA reinforced square laminated composite plate subjected to low velocity central impact (Point A) and eccentric impact (Point B/C/D/E). Here, the above selected moments are on basis of the maximum Mises stress of the square composite laminate subjected to different impact locations. Comparing the five graphs in Figure 8, the maximum Mises stress and the minimum Mises stress value of the laminate in Figure 8b under the eccentric impact point B are higher than that of several other impact cases, which is consistent with the trend of absorption energy curve of the laminate in Figure 7b. The rules are the same. That is to say, the damage of the laminate under the eccentric impact point B is minimal.

#### 4.2.3. Effects of Indenter on Lateral Deflections of Different Points of SMAs/Square Plate

**Figure 9:**
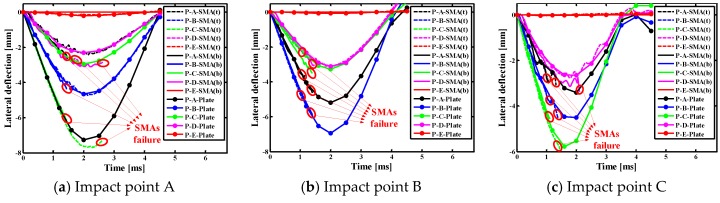

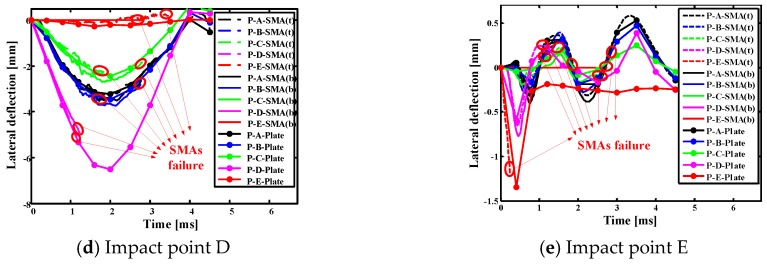
Lateral deflection histories curves of different points of the SMAs/square plate subjected to low velocity central impact (Point A) and eccentric impact (Point B/C/D/E).

Figure 9 show the lateral deflection curves of different points of SMAs/square composite plate subjected to low velocity central impact (Point A) and eccentric impact (Point B/C/D/E). It is worth noting that P-A/B/C/D/E-SMA(t) indicate the point at the A, B, C, D and E positions on the top SMA in the laminate, P-A/B/C/D/E- SMA(b) indicate the point at the A, B, C, D and E positions on the underlying SMA in the laminate, P-A/B/C/D/E-Plate indicate the A, B, C, D and E positions on the uppermost laminate. It can be seen from Figure 9 that the changes of lateral displacement on alloy or plate at the same point may be inconsistent under the center impact (point A), such as the point A and E positions. The changes of the lateral displacement on alloy or plate at the same point are basically consistent under the eccentric impact (point B/C/D/E). In addition, it can be found that the lateral displacement at the impact point on the plate is greatest, whether it is a center impact or an eccentric impact. Comparing the five graphs in Figure 9, the lateral displacement on the alloy and plate is larger when the A, B, and D positions are applied, and that when the C position is applied is follow, and that when the E position is applied is the smallest. This is because the closer the distance between the impact point and boundary, the greater the stiffness of the laminate. It is also concluded that the closer the impact point is to the boundary, the smaller of the effect of the super-elastic performance of the SMA, and the weaker of the effect of the alloy on reducing the deformation of the laminate.

### 4.3. Low Velocity Impact of Rectangular Plates

#### 4.3.1. Typical Impact Curves of Indenter

**Figure 10:**
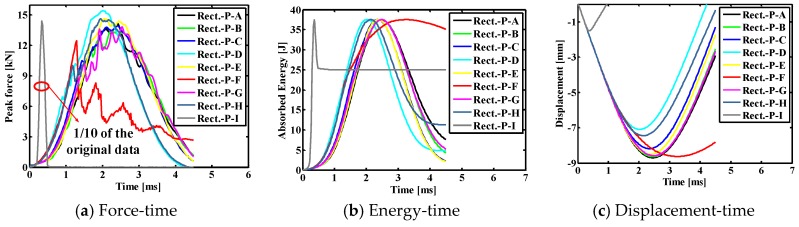
Comparison results between different impact positions of the SMA reinforced rectangular laminated composite plates with stacking sequences [SMA/0_2_/90_2_/0_2_/SMA].

Figure 10 shows the comparison results for the contact force histories, the absorbed energy histories and the displacement histories of indenter of the SMA rectangular laminated composite plate with stacking sequences [SMA/0_2_/90_2_/0_2_/SMA] for the cases of the different impact positions. Here, the contact time between the impactor and the laminate is the longest, the maximum contact force is the smallest, and the displacement value is the largest under center impact (point A). The contact time between the impactor and the laminate is shorter, the maximum contact force value is larger, and the displacement value is smaller under the eccentric impact (point B/C/D/E/F/G/H). The contact time between the impactor and the laminate is the shortest, the maximum contact force value is the largest, and the displacement value is the smallest under the eccentric impact (point I). In addition, the final absorbed energy value from large to small is followed by the condition with impact point F to 1, I, A, G, D, B, H, C, and E. Comparing point A with points B, C, E, and G, it is known that as the distance from the impact point to the boundary decreases, the final absorption energy of the laminate decreases, and the recoverable absorption energy of the entire plate increases. Comparing point A with points F and H, it is known that as the distance from the impact point to the boundary decreases, the final absorption energy of the laminate increases, and the recoverable absorption energy of the entire plate decreases. That is to say, for the rectangular laminates simulated in this paper, the impact resistance of the laminate is better when the impact position is C (37.5, 3, 37.5) and E (25, 3, 75).

In order to more clearly illustrate the peak contact force, the final absorbed energy, and the maximum displacement, the specific values of the impact parameters under different impact positions in the rectangular composite laminate are listed in Table 6. The peak contact force, final absorption energy and maximum displacement value of the composite laminate under the center impact (point A) are 14.15 kN, 7.60 J and −8.70 mm, respectively. The peak contact forces of composite laminates under the eccentric impact (points B/C/D/E/F/G/H/I) are 13.45, 13.91, 15.41, 14.63, 12.46, 13.82, 14.61 and 143.92 kN, respectively, the final absorption energy is 4.24, 2.21, 4.85, 2.08, 35.12, 5.25, 11.30 and 24.98, respectively, the maximum displacement is −8.64, −8.18, −7.10, −8.51, −8.63, −8.59, −7.45 and −1.52 mm, respectively. For the eccentric impact of point I, the peak contact force, the final absorbed energy and the maximum displacement value are quite different from that of other eccentric impact points, this is mainly due to the distance from the impact point to the boundary being smaller than the impactor radius, and the impactor rebound having occurred during the process.

#### 4.3.2. Damage Patterns of SMA Reinforced Rectangular Laminated Composite Plate

**Figure 11:**
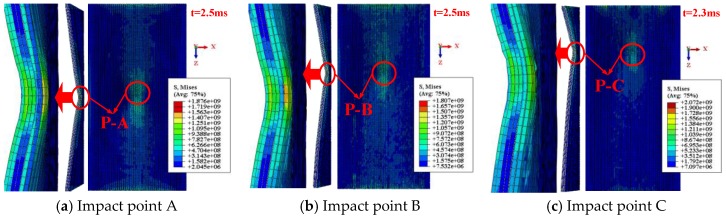

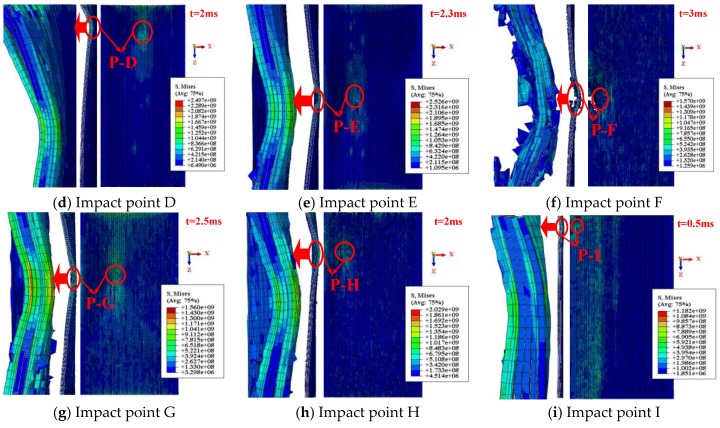
Impact contact surface and cross section damage patterns of SMA reinforced rectangular laminated composite plate subjected to low velocity central impact (Point A) and eccentric impact (Point B/C/D/E/F/G/H/I).

Figure 11 shows the impact contact surface and the cross section damage patterns of SMA reinforced rectangular laminated composite plate subjected to low velocity central impact (Point A) and eccentric impact (Point B/C/D/E). Here, the above selected moments are on basis of the maximum Mises stress of the rectangular composite laminate subjected to different impact locations. Comparing Figure 11, it can be seen that the moment when the maximum Mises stress occurs in the damage diagram of the laminate is the same and their maximum Mises stress value is close under impact points A, B and G, and that is the same and close under impact points C and E, and that is the same and close under impact points D and H. This means that as long as the distance from different impact point to boundary is kept within a certain range, the moment when the maximum Mises stress appears in the damage diagram of the laminate is not greatly affected, and the magnitude of the maximum Mises stress value is not greatly affected. Besides this, when the laminate is applied to the eccentric impact point F, the Mises stress of the laminate is smaller, and the multilayer fibers of the laminate at the impact position are penetrated, and the damage of the laminate is serious, which is consistent with the absorption energy curve of the laminate in Figure 10b.

#### 4.3.3. Effects of Indenter on Lateral Deflections of Different Points of SMAs/Rectangular Plate

**Figure 12:**
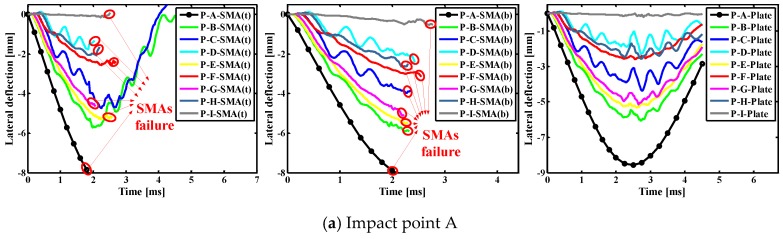

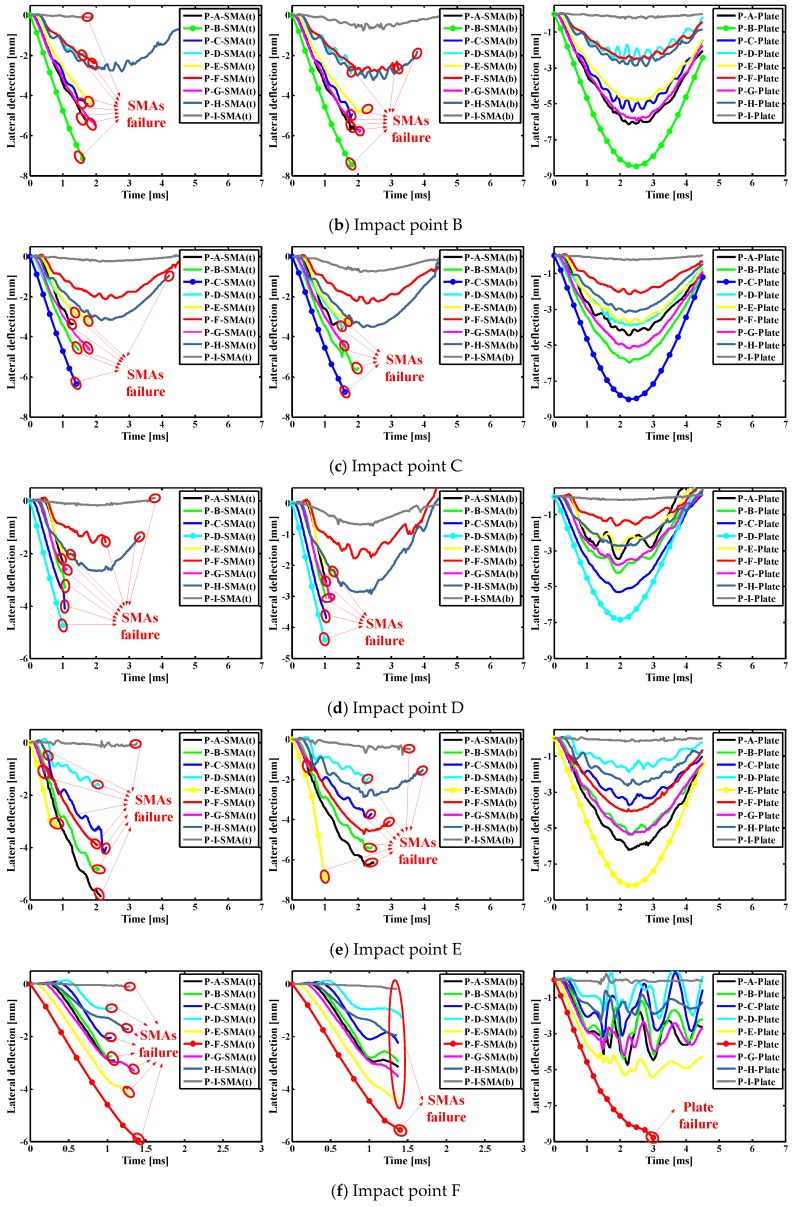

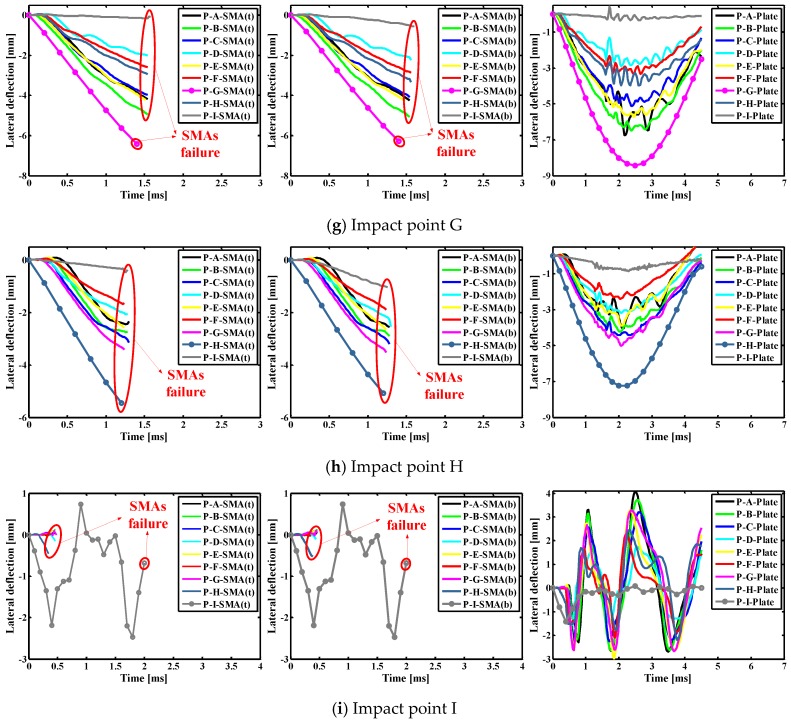
Lateral deflection histories curves of different points on the SMAs/rectangular plate subjected to low velocity central impact (Point A) and eccentric impact (Point B/C/D/E/F/G/H/I).

Figure 12 show the lateral deflection curves of different points on the SMAs/rectangular composite plate subjected to low velocity central impact (Point A) and eccentric impact (Point B/C/D/E/F/G/H/I). It is worth noting that P-A/B/C/D/E/F/G/H/I(t) indicate the point at the A, B, C, D, E, F, G, H and I positions on the top SMA in the laminate, P-A/B/C/D/E/F/G/H/I(b) indicate the point at the A, B, C, D, E, F, G, H and I positions on the underlying SMA in the laminate, P-A/B/C/D/E/F/G/H/I-Plate indicate the A, B, C, D, E, F, G, H and I positions on the uppermost laminate. It can be seen from Figure 12 that the changes of lateral displacement on alloy or plate at the same point are basically consistent under the central impact (point A) and the eccentric impact (point B/C/D/E/F/G/H/I). In addition, it is found that the lateral displacement at the impact point on the plate is greatest, regardless of whether it is a center impact or an eccentric impact. Comparing the five graphs in Figure 12, the lateral displacement on the alloy and plate is larger when A, B, C, E, F and G positions are applied, and that when D and H positions are applied follows, and that when I position applied is the smallest. This is because the closer impact point is to boundary, the greater the stiffness of the laminate. It also can be concluded that the closer the impact point is to the boundary, the smaller of the effect of the super-elastic performance of the SMA, and the weaker of the effect of the alloy on reducing the deformation of the laminate.

## 5. Conclusions

The present numerical results show a general agreement with the results obtained by Shariyat et al. [14]. Based on the numerical simulation results and the corresponding analysis, we may draw the following conclusions:

(1) In general, in terms of the angle of the contact time, contact force, and displacement between the impactor and the laminate, the contact time is shorter, the maximum contact force is larger, and the maximum displacement is smaller under the eccentric impact. This is because the center impact point is the point farthest from the boundary of the laminate, and as the distance decreases, the stiffness of the laminate increases.

(2) A reasonable eccentric impact position is beneficial to reduce the final absorption energy of the laminate and thereby increase the recoverable absorption energy of the entire plate. For all points selected in the square laminate in this paper, the impact resistance of the laminate is better when the impact position is between point B and point D, that is, the z-axis distance is between 21 mm and 25 mm. For all points selected in the rectangular laminate, the impact resistance of the laminate is better when the impact position is points C (37.5 mm, 3 mm, 37.5 mm) and E (25 mm, 3 mm, 75 mm).

(3) In square and rectangular composite laminates, the closer the impact point is to the boundary, the smaller the effect of the superelastic properties of the SMA, and the weaker the effect of the alloy on reducing the deformation of the laminate.

## Figures and Tables

**Table 1:** The material properties of the Ni-Ti shape memory alloy wires.

Parameters	Symbol	Value	Unit
Elastic (Young’s) modulus of austenite/martensite	$E_{A}$, $E_{M}$	51.7, 47.8	GPa
Shear Modulus	G	29.4	GPa
Possion’s ratio of austenite/martensite	$\nu_{A}$, $\nu_{M}$	0.3, 0.3	-
Mass density	ρ	6450	kg/m^3^
Austenitic start/finish temperature at zero stress	$A_{s}$, $A_{f}$	34.5, 49	°C
Martensitic start/finish temperature at zero stress	$M_{s}$, $M_{f}$	18.4, 9	°C
Stress influence coefficient of austenite/martensite	$C_{A}$, $C_{M}$	6.53, 6.53	MPa/°C
Reference temperature	$T_{0}$	37	°C
Maximum recoverable strain	$\varepsilon_{L}$	0.063	-
Initiation/completion stress for transformation into martensite	$\sigma^{Ms}$, $\sigma^{Mf}$	600, 670	MPa
Initiation/completion stress for transformation into austenite	$\sigma^{As}$, $\sigma^{Af}$	288, 254	MPa

**Table 2:** Material properties of the glass fiber-epoxy laminates.

Parameters	Symbol	Values	Units
Young’s modulus	$E_{1}$, $E_{2}$, $E_{3}$	32.062, 10.789, 10.789	GPa
Poisson’s ratio	$\upsilon_{12}$, $\upsilon_{13}$, $\upsilon_{23}$	0.344, 0.344, 0.344	-
Shear modulus	$G_{12}$, $G_{13}$, $G_{23}$	11.92, 11.92, 4.68	GPa

**Table 3:** The strength parameters adopted in FE simulation.

Parameters	Symbol	Values	Units
Ultimate tensile stress	$X_{T}$, $Y_{T}$, $Z_{T}$	1800, 450, 450	MPa
Ultimate compressive stress	$X_{C}$, $Y_{C}$, $Z_{C}$	1800, 450, 450	MPa
Ultimate shear stress	$S_{12}$, $S_{13}$, $S_{23}$	500, 550, 500	MPa

**Table 4:** The values from Shariyat et al. [14] results and present results.

Parameters	Shariyat et al. Results			Present Results		
	A	B	C	A	B	C
Peak force (kN)	13.8	14.6	17	14.14	15.79	17.48
Absorbed energy (J)	7.62	4.37	7.44	6.17	1.79	6.74
Max. displacement (mm)	−7.2	−6.8	−5.4	−7.51	−7.10	−6.01

**Table 5:** Impact parameters under different impact positions in square composite laminates.

Impact Positions	Peak Force (kN)	Absorbed Energy (J)	Max. Displacement (mm)
P-A	14.14	6.17	−7.15
P-B	15.79	1.79	−7.10
P-C	17.48	6.74	−6.01
P-D	16.47	4.04	−6.66
P-E	168.07	19.25	−1.45

**Table 6:** Impact parameters under different impact positions in rectangular composite laminates.

Impact Positions	Peak Force (kN)	Absorbed Energy (J)	Max. Displacement (mm)
P-A	14.15	7.60	−8.70
P-B	13.45	4.24	−8.64
P-C	13.91	2.21	−8.18
P-D	15.41	4.85	−7.10
P-E	14.63	2.08	−8.51
P-F	12.46	35.12	−8.63
P-G	13.82	5.25	−8.59
P-H	14.61	11.30	−7.45
P-I	143.92	24.98	−1.52
